# 2386. Examining Ways to Increase COVID-19 Vaccine Uptake through Motivational Interviewing

**DOI:** 10.1093/ofid/ofad500.2006

**Published:** 2023-11-27

**Authors:** Summer M Reyes, Sydney A Reyes, Tyler Walsh, Jamee T SHELLEY, Humza Agha, Ana V Torres, Jasmine Prater, Gabrielle D Smith, Nicholas Barksdale, Ryan R Lindsay, Sara Malone, Jason Newland

**Affiliations:** Washington University School of Medicine, Saint Louis, Missouri; Washington University in St. Louis School of Medicine, St. Louis, Missouri; Washington University School of Medicine in Saint Louis, St. Louis, Missouri; Washington University School of Medicine, Saint Louis, Missouri; Washington University in St. Louis School of Medicine, St. Louis, Missouri; Washington University in St. Louis School of Medicine, St. Louis, Missouri; Washington University School of Medicine, Saint Louis, Missouri; Washington University in St. Louis School of Medicine, St. Louis, Missouri; Washington University in St. Louis School of Medicine, St. Louis, Missouri; Washington University in St Louis, Saint Louis, Missouri; Washington University School of Medicine, Saint Louis, Missouri; Washington University School of Medicine, Saint Louis, Missouri

## Abstract

**Background:**

COVID-19 vaccine uptake in the U.S. has been poor especially among children. We are conducting a study evaluating 2 theoretically informed strategies to increase COVID-19 vaccine uptake.

**Methods:**

A sequential multiple assignment randomized trail (SMART) design is being conducted where participants are randomized into receiving a text message with a link to COVID-19 vaccine resources or a motivational interview (MI). After 2 weeks, if the person is still not vaccinated, they are re-randomized to one of the same two strategies. Participants were eligible if they were completely unvaccinated or not up to date with their COVID-19 vaccines AND were a resident of St. Louis City or St. Louis County OR a school community member in one of our partnering school districts.

**Results:**

171 individuals have participated in the trial. The median age was 28 (IQR 12-42) Of these, 107 were Black (63%), 44 White (26%), and 114 (67%) female (Table 1). 130 (76%) participants had received the flu vaccination before while only 42 (25%) had received at least one COVID-19 vaccine. A majority (65%) had private insurance; however, 3% had no insurance. Of those who received MI, the average response for importance (1= least, 10=most) of general vaccines and COVID-19 vaccines to one’s health was 7.86 and 4.06, respectively (Table 2). Ten was the most common response of participants for importance of general vaccines and 0 for importance of COVID-19 vaccines and readiness overall. Compared to COVID-19 vaccines, general vaccines had a higher importance overall and at intervention 1 and 2 time points (Figure 1a, b, & c). The four individuals received a COVID-19 vaccine during the study all received a MI call (3 had call as their only intervention while 1 had call then text).

**Table 1:**
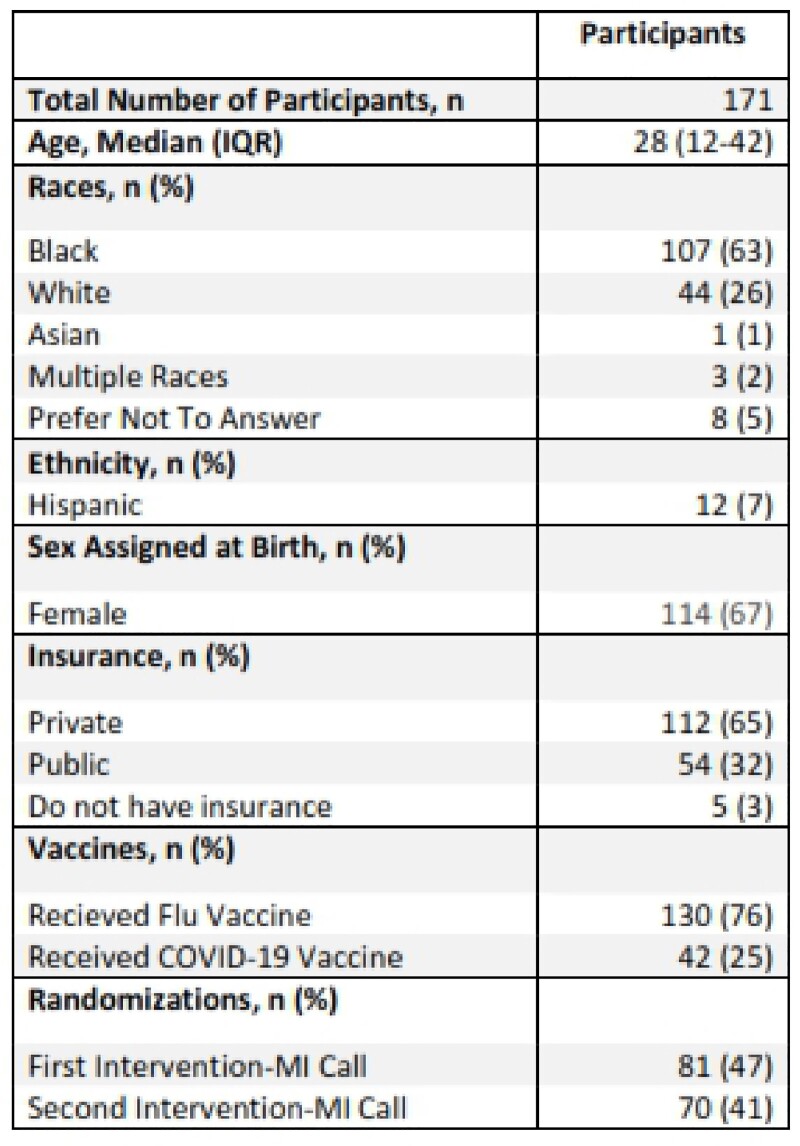
Participant Demographics

**Table 2:**
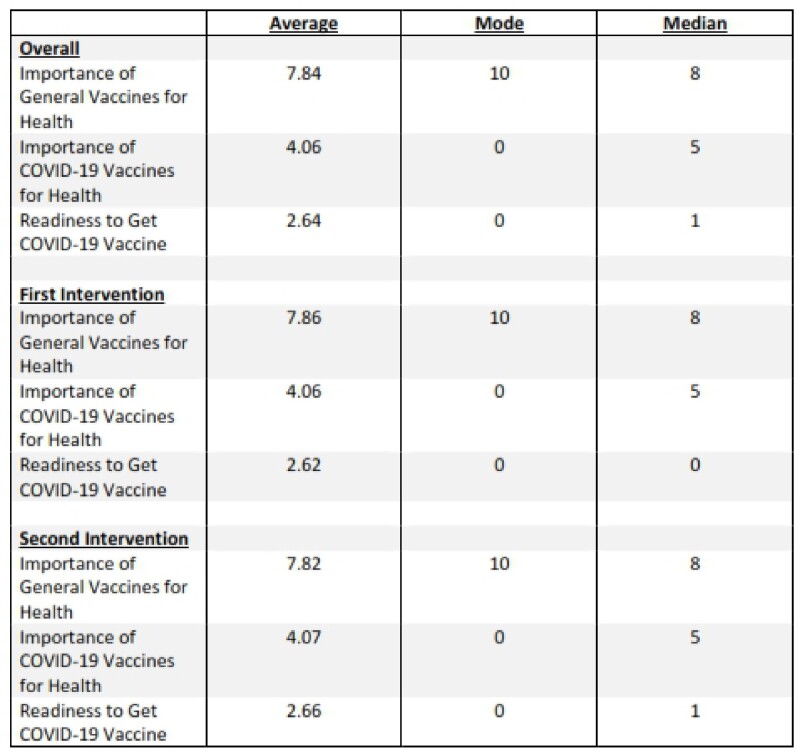
Motivational Interviewing Calls-Overall Numbers

**Figure 1:**
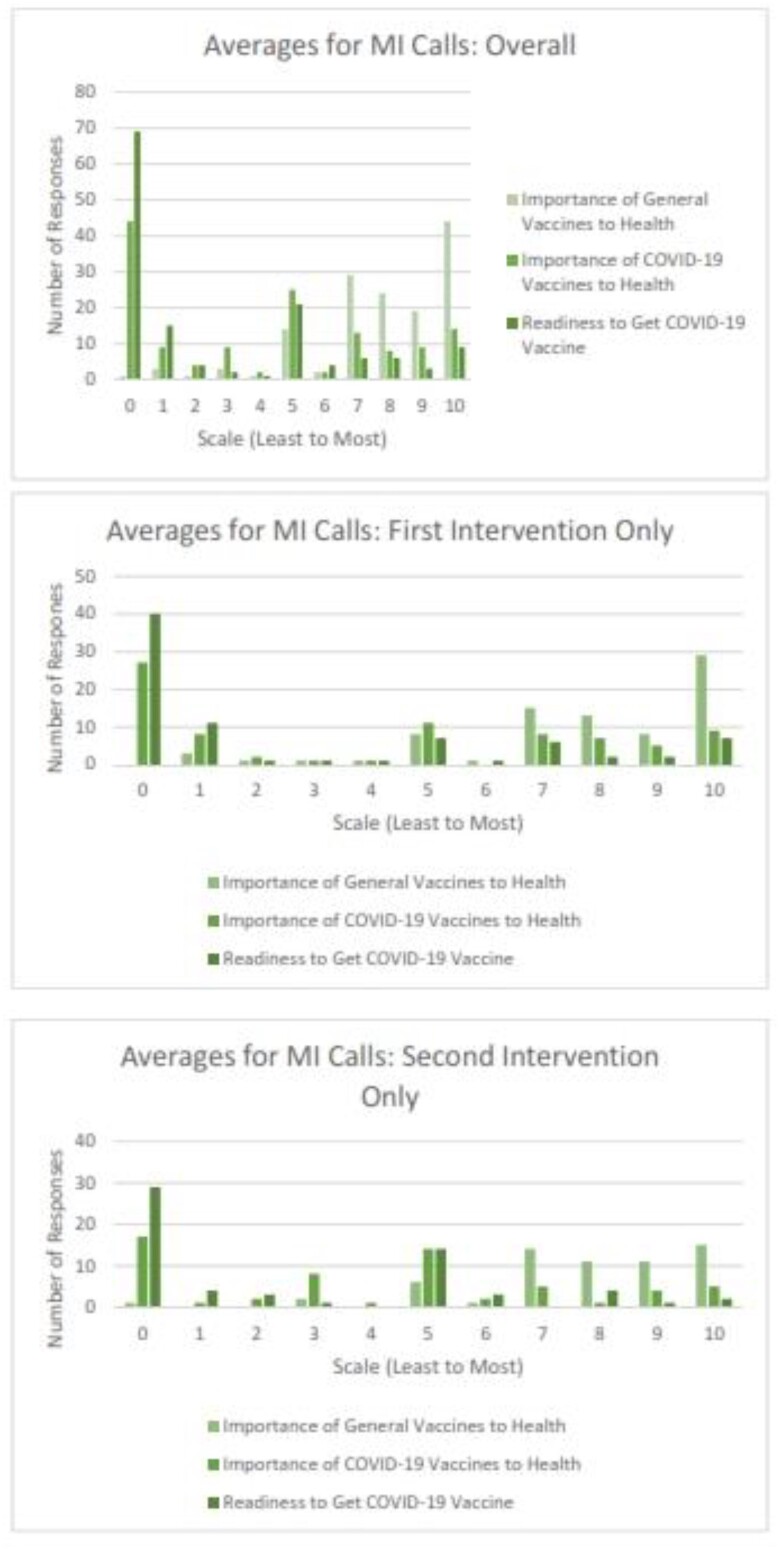
Average Numbers for MI Calls-Overall, First Intervention Only, and Second Intervention Only

**Conclusion:**

General vaccines were regarded more highly than COVID-19 vaccines. With the continuation of this study as well as further analysis in participants’ reasons for their numerical responses, we can further understand why participants view these vaccines differently, what can be done to improve thoughts on COVID-19 vaccines, and increase COVID-19 vaccine uptake.

**Disclosures:**

**Jason Newland, MD**, Moderna: Grant/Research Support|Pfizer: Grant/Research Support

